# Studies of the structure-antioxidant activity relationships and antioxidant activity mechanism of iridoid valepotriates and their degradation products

**DOI:** 10.1371/journal.pone.0189198

**Published:** 2017-12-12

**Authors:** Feifei Wang, Yumei Zhang, Shouhai Wu, Yi He, Zhong Dai, Shuangcheng Ma, Bin Liu

**Affiliations:** 1 School of Chinese Materia Medica, Beijing University of Chinese Medicine, Beijing, China; 2 National Institutes for Food and Drug Control, Beijing, China; 3 The Second Affiliated Hospital, Guangzhou University of Chinese Medicine, Guangzhou, China; Universidade Federal do Rio de Janeiro, BRAZIL

## Abstract

Oxidative stress has been associated with diverse diseases, including obesity, cancer and neurodegeneration. In fact, *Valeriana jatamansi* Jones (valerian) and its extracts possess strong antioxidant activities that extend their application in clinical practice to the treatment of these illnesses, even though the underlying mechanisms are not well understood. Iridoid valepotriate, a characteristic iridoid ester in valerian with poor chemical stability, possesses considerable antioxidant components. The original compounds and their degradation products have been found to exhibit strong antioxidant activities. However, the relationship between their structure and antioxidant effects and the mechanism underlying their oxidation resistance remain unclear. A forced degradation study using three iridoid valepotriates (valtrate, acevaltrate and 1-*β* acevaltrate) was performed in this work, and the structures of their degradation products were estimated by TLC-MS and LC-MS. Comparison of the antioxidant activities of the iridoid valepotriates before and after forced degradation revealed that degradation reduced the activities of the iridoid valepotriates in free radical scavenging and cytotoxic and cell apoptosis tests. The results suggested that the oxirane nucleus is important for defining the antioxidant profile of iridoid valepotriate. We uncovered possible mechanisms that could explain the antioxidant activities, including the generation of two hydroxyl groups through intramolecular transfer of an H^•^ from an oxirane ring and a reduction in ROS levels through interactions with GABAergic signalling pathways.

## Introduction

The human body constantly reacts with oxygen to produce highly reactive molecules known as reactive oxygen species (ROS) or free radicals. The strong cellular oxidizing potential of excess ROS might cause oxidative damage to proteins, membranes and genes [1, 2]. Oxidative stress has been implicated as a major cause of cellular injuries in a wide variety of clinical abnormalities, particularly in the central nervous system (CNS). Conversely, in tumour cells, the generation of ROS has contributed to the development of sophisticated systems to counterbalance oxidative stress through rapid proliferation [3]. Based on this information, drugs with antioxidant activities are widely used for the treatment of cancer, anxiety neurosis, and Parkinson’s disease, among other diseases.

As an important neuroprotective and anticancer plant, *Valerian jatamansi* Jones (valerian) has been extensively used as an herb medicine or dietary supplement. This species has been traditionally used as therapy for sleeping disorders, obesity, epilepsy, insanity, snake poisoning, and eye and skin problems [4,5]. Its antioxidant activities, cytotoxic effects, and anti-leishmanial, anti-Parkinson and anti-HIV activities have been of particular interest recently [4, 6–13]. Valerian also contains various secondary metabolites, including iridoid valepotriates, valerenic acids, flavonoids and tnnins [14].

Iridoid valepotriates, the characteristic iridoid esters in valerian, have attracted great interest due to their various biologically significant properties [15–18]. Previous studies have indicated that iridoid valepotriates display potential dose-dependent oxidation resistance abilities. However, the poor stability of iridoid valepotriate is well-known: it can easily decompose under strong sunlight or under high temperature, acidic or alkaline conditions [19, 20]. Paradoxically, both the natural and decomposition products (baldrinal and homobaldrinal) have multiple biological activities [21–23], but the degradation pathways and antioxidative mechanisms have not yet been systematically documented.

**Fig 1:**
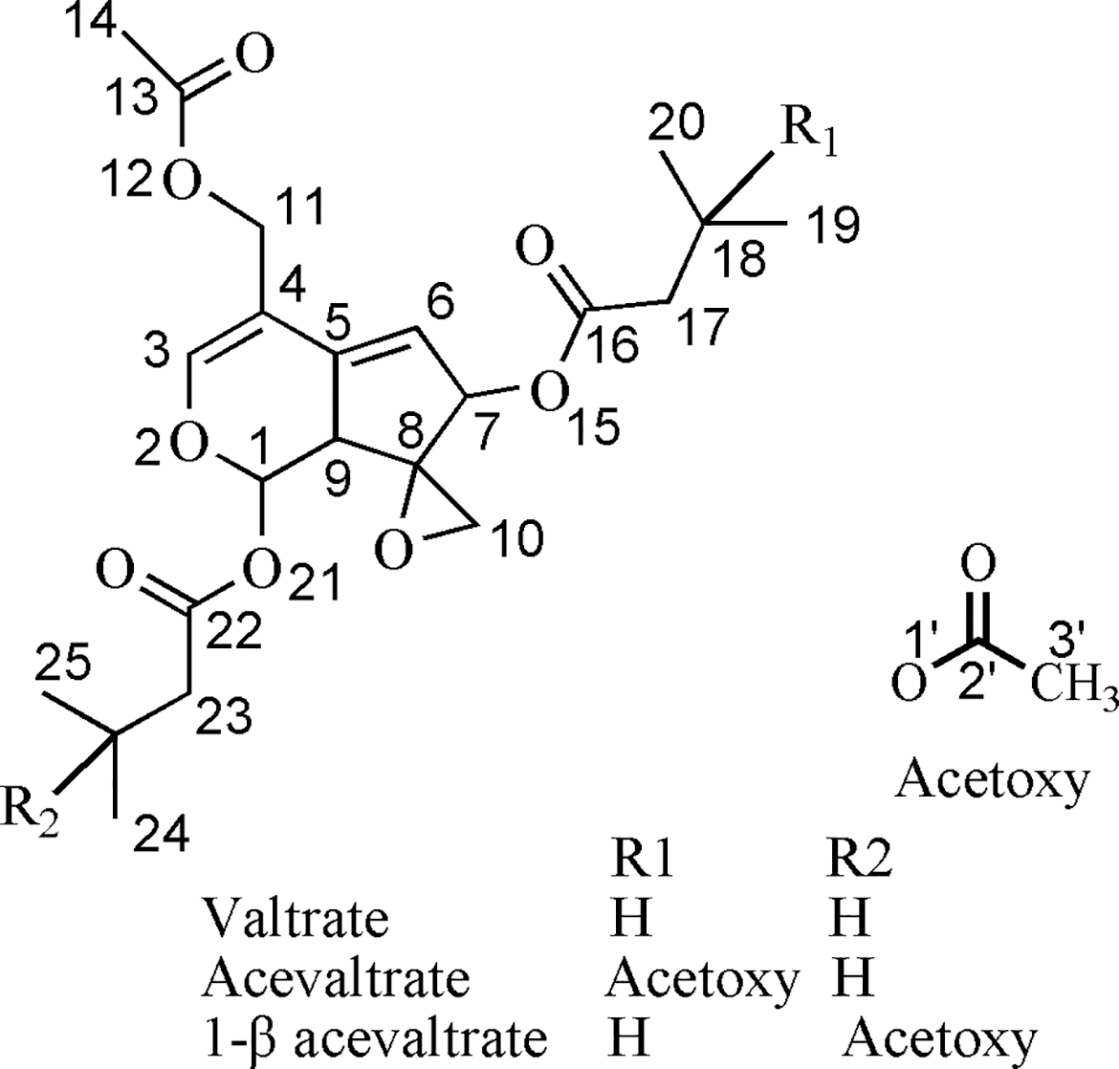
Structural formulas of V, AV and BAV.

We initially identified the structural characteristics of iridoid valepotriates [valtrate (V), acevaltrate (AV) and 1-*β* acevaltrate (BAV), Fig 1] and their degradation products. Iridoid valepotriates are labile to thermal degradation and base hydrolysis. Their structure-activity relationships were explored through an evaluation of their antioxidant effects. Our results demonstrate that iridoid valepotriates could promote cytotoxicity in three cancer cells without affecting non-malignant cells. The stability of the oxirane nucleus was found to be important due to the ability of iridoid valepotriates to donate two hydrogen molecules through intramolecular transfer of H^•^ from the oxirane ring, which might be one mechanism contributing to the antioxidant activity profile of these compounds. Additionally, GABAergic signalling pathways were discussed as another possible pathway through which iridoid valepotriates reduce ROS and induce HepG2 cell apoptosis.

## Materials and methods

### Reagents and samples

HPLC-grade methanol and acetonitrile were purchased from Fisher Scientific (Fair Lawn, NJ, USA). A Milli-Q water purification system (Millipore, San Diego, CA, USA) was used to further purify distilled water. Other materials included cisplatin (CP), N, N- Dimethylformamide (DMF), 2,2’-azino-bis(3-ethylbenzo-thiazoline-6-sulfonic acid) diammonium salt (ABTS), 1,1-diphenyl-2-picrylhydrazyl radical 2,2-diphenyl-1-(2,4,6-trinitrophenyl) hydrazyl (DPPH), 3-(4,5-dimethylthiazole-2-yl)-2,5-diphenyltetrazolium bromide (MTT), dimethylsulfoxide (DMSO), butylated hydroxytoluene (BHT), 2,7-dichlorodihydrofluorescein diacetate (DCFH-DA) and 2,7-dichlorofluorescein (DCF), all of which were purchased from Sigma Aldrich Co. (St. Louis, MO, USA). Annexin V-fluorescein isothiocyanate (Annexin V-FITC) and a propidium iodide (PI) kit were obtained from BD (San Jose, CA, USA). Valtrate (lot: 111840–201402), acevaltrate (lot: 111841–201401) and 1-*β* acevaltrate were obtained from the National Institutes for Food and Drug Control of China. The analytical reagent-grade sodium hydroxide, hydrochloric acid and hydrogen peroxide (H_2_O_2_, 30%) used in this study were purchased from Beijing Chemical Works (Beijing, China).

### General experimental procedures

NMR spectra were obtained using a Bruker Avance-III 600 MHz spectrometer (Bruker Corp, Karlsruhe, Germany) in CD_3_OD with tetramethylsilane (TMS) as the internal standard. The liquid chromatography-mass spectrometry (LC-MS) system (Agilent 6410 Triple Quad, CA, USA; Q-TOF, Waters, USA) was used to analyse the forced degradation products. LC was performed on an Agilent SB-C_18_ column (1 μL/mL, 4.6 mm × 250 mm, 5 μm) at 30°C, and the mobile phase consisted of a mixture of methanol and water (70:30, v/v). Mass spectrometry analyses were performed in the positive ionization mode with a scanning range of *m/z* 80 ~ 1, 000. The following electrospray ionization (ESI) parameters were set: the capillary voltage was 4,000 V with a target mass-to-charge ratio of 150, the nebulizer (N_2_) pressure was set to 30 psi, the drying gas (N_2_) temperature was 325°C, and the drying gas flow rate was 7 L/min. For the MS/MS investigations, the ions of interest were isolated through quadrupole tandem mass spectrometry. Thin-layer chromatography (TLC) was performed on silica gel 60 F_254_ TLC plates (Merck, Darmstadt, Germany) and developed using a cyclohexane:acetone mixture (5: 1, v/v). After drying, the TLC plates were examined under UV light at 254 nm in a TLC visualizer (Camag, Muttenz, Switzerland).

### Stressed degradation conditions

The stress degradation studies of iridoid valepotriates were performed based on the ICH guidelines [24, 25]. The following optimized stress conditions were used: acidic hydrolysis (0.01 M HCl at 25°C for 5 h), alkaline hydrolysis (0.01 M NaOH at 25°C for 10 min), oxidative hydrolysis (3% ~ 6% H_2_O_2_ at 25°C for 24 h), and thermal degradation (menthol solution at 60°C for 0, 1, 2, 4, or 6 h; solids at 4°C for 7 days; solids at 25°C for 7 days). The drugs were exposed to light (intensity of 5000 lx for 3 h) [26] and then dissolved and diluted in menthol to a final concentration of 1 mg/mL (for current use).

### TLC-DPPH^•^ bioautography/MS analysis

For the DPPH^•^ analysis, 2 mL of the drugs at various concentrations was added to 2 mL of DPPH^•^ solution (0.1 mM, 2 mL). After 20 min of incubation at 37°C in the dark, the absorbance was recorded at 517 nm using a UV-Visible spectrophotometer (Evolution 260 Bio, Thermo Scientific, USA).

The TLC-DPPH^•^ assay was conducted as a screening test to estimate the antioxidant properties of the tested fractions [27, 28]. Two TLC plates were developed in parallel. The TLC operation was completed as described in the ‘General experimental procedures’ section. After development and drying, one TLC plate was examined under UV light at 254 nm; the fluorescence-quenching spots were marked and then subjected to TLC-MS analysis. The other TLC plate was sprayed with 0.04% DPPH^•^-methanol solution (20 mg of DPPH^•^ dissolved in 50 mL of methanol). The free radical scavenging compounds appeared as yellowish spots on the purple background. The yellowish white bands indicated that those compounds possessed antioxidant activities. The intensity illustrated the antioxidant capacities of the samples.

For the MS analysis, the TLC-MS interface (Camag, Muttenz, Switzerland) inlet capillary was connected to an HPLC pump. The extraction was performed with methanol at a flow rate of 0.1 mL/min and directly connected with the ESI-MS instrument. The zones were then extracted during a 1.8-min time period with an oval extraction plunger and individually transferred into the ESI-MS instrument. The plunger head was cleaned with N_2_ gas for 4 s between the extraction steps.

### ABTS radical scavenging activity

The reaction mixture comprised 950 μL of ABTS solution and 50 μL of the samples at various concentrations in a final volume of 1 mL. The reaction mixture was homogenized and incubated for 20 min. The absorbance of the solutions at 734 nm was measured with UV-Visible spectrophotometer.

### Cytotoxicity assay

#### Cell lines and culture

Human liver carcinoma (HepG2) cells, human cervix carcinoma (HeLa) cells, breast cancer (MDA-MB-231) cells and human umbilical vein endothelial cells (HUVECs) were obtained from Zhongshan University, Guangzhou, China. All of the cells were cultured in Dulbecco’s modified Eagle media (DMEM) supplemented with 10% foetal calf serum, 100 units/mL penicillin G and 100 μg/mL streptomycin. All media and serum were obtained from Gibco (USA). The cells were grown and maintained at 37°C in a 5% CO_2_ humidified atmosphere.

#### Cytotoxicity analysis via MTT assay

The cytotoxicities of the drugs and their degradation components (stored at 25°C for 7 day) were evaluated through a modified MTT assay [29]. The cells were plated at a concentration of 10^4^ cells/well in 96-well plates (Corning, Sigma, USA) containing 100 μL of growth medium per well for 24 h. The stock solution was prepared by dissolving 10 mg of the sample in 1 mL of DMSO (AR). The stock solution was then diluted with DMSO to produce solutions with different concentrations. Finally, 1 μL of each of the solutions was added to 100 μL of growth medium in 96-well plates to produce sample solutions with concentrations ranging from 10 to 100 μM. No precipitation was observed in each of the solutions by visual inspection, which suggested that the chemical compounds were completely dissolved in the solvent. After 24 h of incubation, the cells were treated with 20 μL of MTT (5 mg/mL) and incubated at 37°C for 4 h. The medium was removed, and 100 μL of DMSO was added to each well. After shaking for 10 min, the absorbance at 490 nm was measured. The mitochondrial activity related to viability is expressed as the concentration of drug that inhibited cell growth by 50% (IC_50_) compared with the negative control (cells treated with 1.0% DMSO) and the positive control (serially diluted cisplatin solution). The tests and analyses were run in triplicate (n = 3), and averages of the results are reported.

#### Flow cytometry analysis for ROS measurements

DCFH-DA was used for ROS capture in the cells. The average fluorescent intensity of DCF is proportional to the intracellular ROS levels. HepG2 cells were seeded in 6-cm dishes and treated with the drugs. Twenty-four hours after treatment, the HepG2 cells (approximately 10^5^ cells) were washed, resuspended in PBS and treated with DCFH-DA at a final concentration of 10 μM. The cells were incubated at 37°C for 30 min in the dark, and the ROS levels were then measured by flow cytometry (BD, San Jose, CA, USA). At least 20,000 events were analysed.

#### Flow cytometry analysis for apoptosis assessment

The cells were treated with the drugs for 24 h, washed twice with pre-chilled PBS and then re-suspended in 100 μL of binding buffer. The fraction of apoptotic cells was assessed via flow cytometry using an Annexin V-FITC/PI kit. The drugs were prepared according to the manufacturer’s instructions and measured by flow cytometry analysis. At least 20,000 events were analysed.

### Statistical analyses

SPSS 17.0 software was used for the statistical analyses. The data are presented as the means ± SDs. Analysis of variance was applied for the comparisons of the means among two or multiple groups. ANOVA was further used for comparisons between two groups. The differences between samples were statistically significant at *p < 0.05.

## Results and discussion

### Structural identification of AV and BAV

AV and BAV are isomers, even though their structures have been confused in the literature [7, 30]. Thus, LC-MS, UV and NMR analyses were performed to confirm their chemical structures. Heteronuclear multiple bond coherence (HMBC) and heteronuclear multiple quantum coherence (HMQC) studies were conducted to confirm the acyl groups’ chemical shift data (S1 Fig). The acyl groups, which connect to different positions on the iridoid rings, showed chemical shift differences of 0.2–0.3 ppm in their ^13^C NMR spectra (the *γ*-effect) [6, 31, 32]. Based on the HMBC data, the chemical shifts of the four ester carbonyls were assigned as *δ*_C_ 172.6 (C-13), 171.2 (C-16), 171.8 (C-22) and 172.2 (C-2’) for AV and *δ*_C_ 172.6 (C-13), 172.9 (C-16), 168.9 (C-22) and 172.3 (C-2’) for BAV; these results correspond to the data in the literature [32,33] (S1 Fig).

### Characterization of the V, AV, BAV and their degradation products

**Fig 2:**
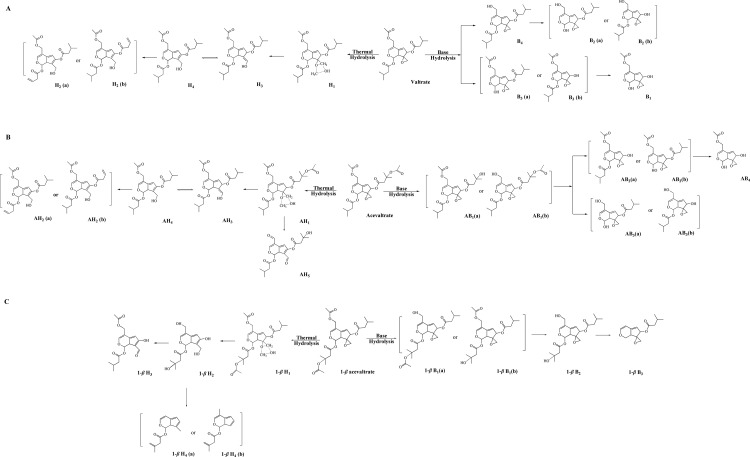
Schematic representation of acevaltrate degradation (AVD).

Iridoid valepotriates are thermolabile and decompose easily under acid or alkaline conditions or in alcoholic solutions [34]. In this work, forced degradations (e.g., acid, alkaline, oxidation, photolysis and thermal degradation) were performed to obtain the desired compounds. The degradation pathways were estimated by LC-MS analysis (Fig 2).

In the LC-MS chromatogram obtained during alkaline hydrolysis, a total of four AV degradation components were detected and identified (Fig 2B). AV showed a pseudo-molecular ion at *m/z* 503 [M+Na] ^+^, and the MS/MS spectrum of the *m/z* 503 ion showed product ions at *m/z* 219, 177, 149 and 131 (S2 Fig). The degradation products from alkaline hydrolysis showed the same UV maximum absorption at approximately 256 nm, suggesting that the iridoid ring was stable during degradation [35].

AB_1_, the main alkaline degradation product of AV, showed a pseudo-molecular ion at *m/z* 461 (C_22_H_30_O_9_Na) in the MS spectra, which suggested that the compound was produced by cleavage of the acetyl group at AV O-1’ or O-12. The MS/MS spectra of AB_1_ and AB_2_ had almost identical predominant product ions at *m/z* 255 [M-C_5_H_9_O_2_-C_5_H_9_O]. As a consequence, AB_2_ was identified as a basic degradation product of AB_1_ with the pseudo-molecular ion at *m/z* 361 (C_17_H_22_O_7_Na) due to loss of a C_5_H_9_O_2_ group from AB_1_. AB_3_ had a pseudo-molecular ion at *m/z* 319 (C_15_H_20_O_6_Na), which is 42 Da less than that of AB_2_ due to cleavage of an acetyl group. The predominant product ion in the AB_3_ MS/MS spectrum was located at *m/z* 277 [M-H_2_O]. The MS spectrum of AB_4_ showed a pseudo-molecular ion at *m/z* 277 (C_15_H_20_O_6_Na), indicating that AB_4_ was the hydrolysis product of AB_2_ generated by loss of a C_5_H_9_O group.

A total of five degradation components were identified in the AV thermal solution obtained under the degradation condition. The thermal degradation products AH_1_-AH_4_ showed a maximum UV absorption at approximately 240 nm, which might be the result of a ring-opening reaction [10]. The MS spectrum of AH_1_ showed a pseudo-molecular ion at *m/z* 535 (C_25_H_36_O_11_Na), and the daughter ions of the pseudo-molecular ion were located at *m/z* 251 and 191. The AH_1_, AH_2_ and AH_4_ degradation products were all formed by opening of the oxirane ring and possessed similar MS/MS spectrums, including daughter ions at *m/z* 305, 233 and 191. AH_3_ *m/z* 423 (C_22_H_30_O_8_H) and AH_4_ *m/z* 423 (C_22_H_30_O_8_H) were obtained by loss of a C_3_H_6_O_3_ group from AH_1_. AH_4_ (hydroxymethyl formation of V) and AH_5_ *m/z* 393 (C_20_H_24_O_8_H) displayed ions at *m/z* 293 [M-H_2_O-C_5_H_9_O]. The maximum UV absorption wavelength of AH_5_ was 263 nm, which suggested that formation of an aldehyde group in iridoid valepotriate could reinforce the conjugated system. AH_5_ was obtained by the loss of two oxoethyl groups from at O-12 and O-2’ positions of AV.

Iridoid valepotriates were stable under light (3 h) and thermal degradation (solid at 60°C for 24 h) conditions. The degradation products in the H_2_O_2_ solution (24 h) had no typical LC profiles. The degradation products obtained by acidic hydrolysis were similar to those of the base reaction. V and BAV displayed comparable degradation pathways to AV (Fig 2A and 2C), although the stability of BAV was notably lower than those of V and AV.

### Analysis of antioxidant activities

To evaluate the antioxidant activities, we assessed their performances in ABTS and TLC-DPPH^•^ bioautography/MS assays. Alkaline hydrolysis is strongly exothermic, and the solution obtained from this reaction turns black within 10 min. The degradation products obtained through thermal degradation (methanol solution at 60°C for 1, 2, 4, or 6 h; methanol solution at 25°C for 7 days) were tested through TLC-DPPH^•^ bioautography assay.

### TLC-DPPH^•^ bioautography/MS analysis

The original compounds showed good separation from their degradation products by TLC (S3 Fig). In the chromatograms, the yellow spots for V, AV and BAV appeared immediately after DPPH^•^ staining on the silica gel, whereas light yellow spots for the degradation products appeared slowly.

The structures of the original compounds and their degradation products were subsequently confirmed by “dot-blot” MS analysis. The structures of the degradation compounds estimated from the TLC-DPPH^•^ bioautography/MS analysis compared well with those obtained with the LC-MS analysis. The MS data and identified compounds are shown in Table 1.

**Table 1:** MS and MS/MS spectroscopic data for the iridoid valepotriates and their degradation products.

	Compound	HPLC-MS	TLC-MS	${UV}_{\lambda_{nm}^{max}}$	MS Spectrum	Formula
**Known compounds**	V	+	+	256	445 [M+Na]^+^	C_22_H_30_O_8_
	AV	+	+	256	503 [M+Na]^+^	C_24_H_32_O_10_
	BAV	+	+	256	503 [M+Na]^+^	C_24_H_32_O_10_
**Alkaline hydrolysis**	B_1_	+	-	256	403 [M+Na]^+^	C_20_H_28_O_7_
	B_2_	+	-	256	361 [M+Na]^+^	C_17_H_22_O_7_
	B_3_	+	-	256	319 [M+Na]^+^	C_15_H_20_O_6_
	B_4_	+	-	256	277 [M+Na]^+^	C_15_H_14_O_6_
	AB_1_	+	-	256	461 [M+Na]^+^	C_22_H_30_O_9_
	AB_2_	+	-	256	361 [M+Na]^+^	C_17_H_22_O_7_
	AB_3_	+	-	256	319 [M+Na]^+^	C_15_H_20_O_6_
	AB_4_	+	-	256	277 [M+Na]^+^	C_12_H_14_O_6_
	1-*β* B_1_	+	-	256	461 [M+Na]^+^	C_22_H_30_O_9_
	1-*β* B_2_	+	-	256	419 [M+Na]^+^	C_20_H_28_O_8_
	1-*β* B_3_	+	-	256	283 [M+Na]^+^	C_14_H_18_O_4_
**Thermal degradations**	H_1_	+	-	254	535 [M+Na]^+^	C_25_H_36_O_11_
	H_2_	+	+	240	423 [M+H]^+^	C_22_H_30_O_8_
	H_3_	+	+	243	423 [M+H]^+^	C_22_H_30_O_8_
	H_4_	+	+	236	407 [M+H]^+^	C_22_H_30_O_7_
	AH_1_	+	+	254	535 [M+Na]^+^	C_25_H_36_O_11_
	AH_2_	+	+	240	423 [M+H]^+^	C_22_H_30_O_8_
	AH_3_	+	+	243	423 [M+H]^+^	C_22_H_30_O_8_
	AH_4_	+	+	236	407 [M+H]^+^	C_22_H_30_O_7_
	AH_5_	+	+	264	391 [M+H]^+^	C_21_H_28_O_7_
	1-*β* H_1_	+	-	254	535 [M+Na]^+^	C_25_H_36_O_11_
	1-*β* H_2_	+	+	245	313 [M+H]^+^	C_15_H_20_O_7_
	1-*β* H_3_	+	+	263	337 [M+H]^+^	C_17_H_18_O_7_
	1-*β* H_4_	+	+	241	232 [M+H]^+^	C_14_H_14_O_3_

+ identified - undetected

### ABTS and DPPH^•^ assays

The solution conditions produced the same degradation products at 60°C (for 1, 2, 4, and 6 h) and 25°C (7 days), and the latter condition was selected for further studies. Antioxidant activities were evaluated through ABTS and DPPH^•^ assays using BHT as a reference or positive control. The concentrations that inhibited 50% of the activity in each test (IC_50_ values) are shown in Table 2. The maximum activity was recorded with V (IC_50_ ranging from 1.6 ± 0.3 μM from the ABTS assay to 3.6 ± 0.2 μM from the DPPH^•^ assay), and the minimum activity was obtained with 1-*β* acevaltrate, a BAV degradation product (IC_50_ ranging from 63.4 ± 1.2 μM from the ABTS assay to 89.4 ± 2.3 μM from the DPPH assay). The activities could be ordered as follows: V > AV > BAV > valtrate degradation products (VD) > AVD > BAVD.

**Table 2:** Antioxidant effect of the iridoid valepotriates assessed through ABTS and DPPT^•^ assays.

Compounds	IC_50_ (μM, n = 3)	
	ABTS	DPPH
**V**	1.6 ± 0.3	3.6 ± 0.2
**AV**	2.5 ± 0.2	5.4 ± 0.1
**BAV**	14.9 ± 0.4	38.4 ± 0.8
**VD**	20.5 ± 2.1	35.5 ± 2.7
**AVD**	29.5 ± 1.5	56.2 ± 1.2
**BAVD**	63.4 ± 1.2	89.4 ± 2.3
**BHT**	28.6 ± 0.8	110.4 ± 1.1

### Cytotoxic tests

#### Effect on cancer cell proliferation

The anti-proliferative effects of V, AV, BAV and their degradation products were investigated on three human cancer cells through an MTT assay (Table 3). The data showed that BAVD exerted relatively weak anti-proliferative effects on the three cancer cell lines. V displayed better anti-proliferative activities, with IC_50_ values ranging from 25.4 ± 0.6 μM (HepG2 cells) to 37.1 ± 1.1 μM (HeLa cells). All of the samples demonstrated relatively high anti-proliferative activities in HepG2 cells.

**Table 3:** Cytotoxicity of iridoid valepotriates against HepG2, HeLa and MDA-MB-231 cells and HUVECs.

Compound	Cancer Cell Lines (IC_50_, μM, n = 3)			
	HepG2	HeLa	MDA-MB-231	HUVEC
**V**	25.4 ± 0.6	37.1 ± 1.1	30.5 ± 0.5	97.5
**AV**	30.4 ± 0.7	42.3 ± 1.3	37.5 ± 0.5	92.2
**BAV**	48.5 ± 2.1	50.3 ± 0.8	60.3 ± 1.8	>100
**VD**	50.5 ± 1.9	60.1 ± 2.1	75.1 ± 1.9	89.1 ± 1.9
**AVD**	62.4 ± 0.9	>100	80.2 ± 1.4	>100
**BAVD**	>100	>100	>100	>100
**CP**	15.2 ± 1.2	12.7 ± 0.6	13.1 ± 1.1	95.6 ± 1.3

HepG2 cells were selected for further mechanistic studies because they were more sensitive to the tested compounds. The MTT results suggested that the iridoid valepotriates were more active against cancer cells than non-malignant cells (HUVECs; IC_50_ = 25.4 *vs*. IC_50_ = 97.5 μM for V). The cells were then treated with the drugs at a concentration of approximately 40 μM for further tests.

### ROS measurements

**Fig 3:**
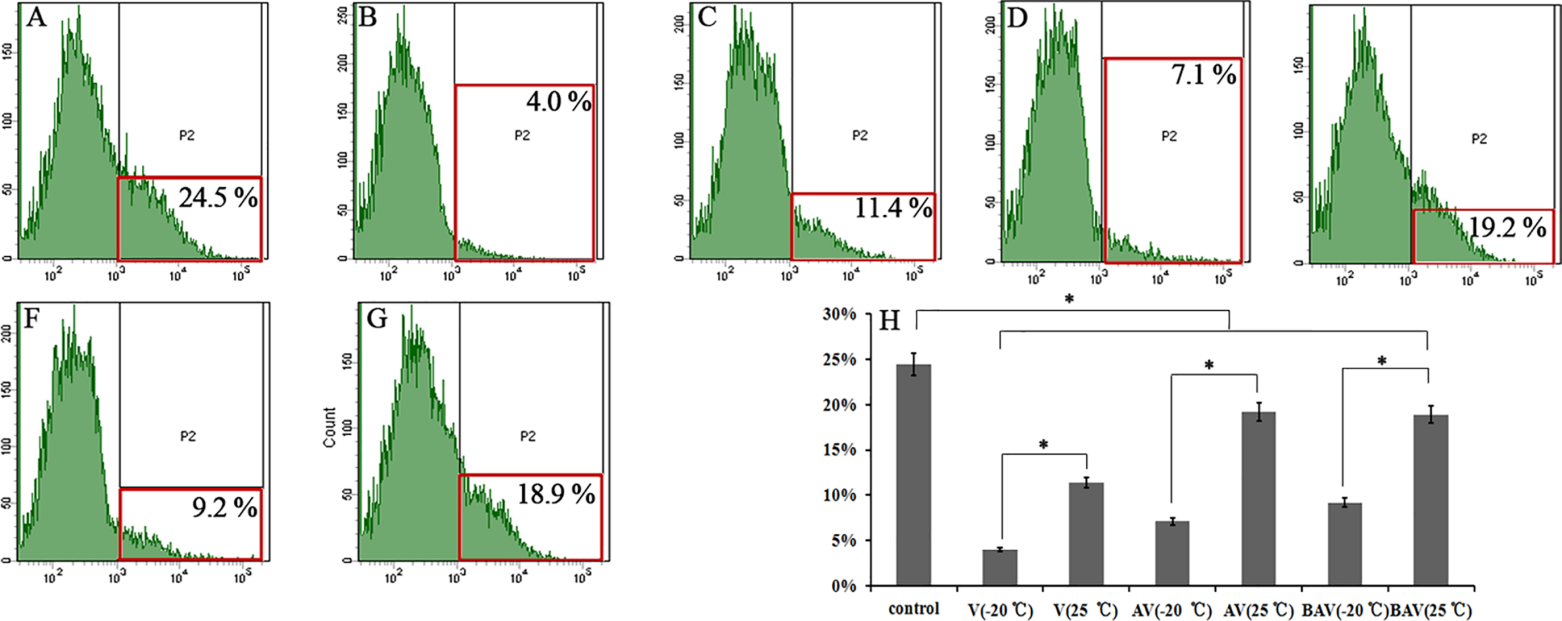
Effect of V, AV, BAV and their degradation products on ROS in HepG2 cells. ROS in the cells was labelled with DCFH-DA and measured by flow cytometry. (A—G) Results for the (A) control, (B) V (-20°C), (C) V (25°C for 7 days), (D) AV (-20°C), (E) AV (25°C for 7 days), (F) BAV (-20°C) and (G) BAV (25°C for 7 days) groups. (H) The data are presented as the means ± SDs from three separate experiments (* *p* < 0.05).

The ROS concentrations in HepG2 cells were measured to determine whether iridoid valepotriates can alter the intracellular ROS levels. The independent experiments were performed in triplicate (n = 3). The ROS levels were determined using the cell-permeable oxidation-sensitive dye DCFH-DA, and the fluorescence in the cells was quantified by flow cytometry. As shown in Fig 3A–3G, the iridoid valepotriates induced a significant decrease in ROS concentrations compared with the untreated control group and the degradation products. The maximum activity was recorded for V (4.0 ± 0.3%), and the minimum activity was obtained with AVD (19.2 ± 0.9%) (Fig 3H).

### Treatment with iridoid valepotriates and their degradation products induced apoptosis in HepG2 cells

Apoptosis tests were performed in HepG2 cells to confirm the anti-apoptotic effects of these compounds. Apoptotic cells were defined as Annexin V-FITC-positive cells and specifically refers to the sum of early (quadrant 4, Q4) and late (Q2) apoptotic cells [36, 37].

**Fig 4:**
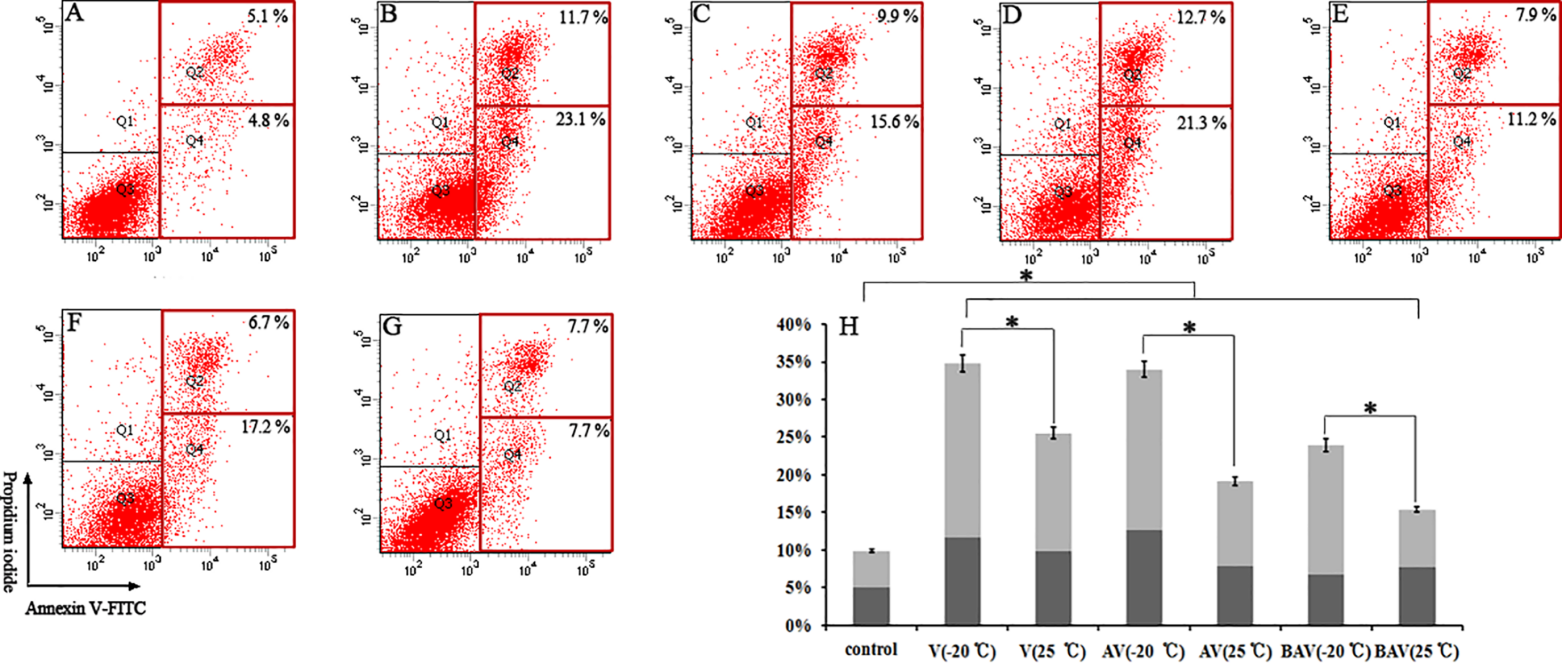
Apoptosis was measured by flow cytometry through Annexin V-FITC and PI staining. Representative plots from one set of Annexin V-FITC flow cytometry and PI staining experiments performed in triplicate are shown. Early apoptotic cells (Annexin-V+ and PI-) are shown in the lower right quadrant, and late apoptotic cells (Annexin-V+ and PI+) are shown in the upper right quadrant. The percentages of apoptotic cells are indicated as Annexin-V+ cells and are shown as the means ± the SD from three independent experiments. (A—G) Results from the (A) control, (B) V (-20°C), (C) V (25°C for 7 days), (D) AV (-20°C), (E) AV (25°C for 7 days), (F) BAV (-20°C) and (G) BAV (25°C for 7 days) groups. (H) The data are presented as the means ± the SD from three separate experiments (* *p* < 0.05).

As clearly shown in Fig 4H, the early and late apoptotic rates were significantly increased (*p < 0.05) in HepG2 cells compared with the control group. The maximum activity was observed with treatment with V (ranging from 4.8 ± 0.9% to 23.1 ± 0.7% for early apoptosis and from 5.1 ± 1.0% to 11.7 ± 0.6% for late apoptosis) (Fig 4B). The other tested compounds also yielded increased apoptosis rates compared with the control group (*p* < 0.05) (Fig 4A–4G).

## Discussion

In this study, forced degradations were performed to assess the behaviours of iridoid valepotriates and their degradation products under various stress conditions. V, AV and BAV were labile to base hydrolysis largely due to their ester functionality [33]. According to the FDA and ICH guidelines and published papers, the desired degradation levels were set to between 15 and 20% degradation in this work [24, 25]. Thus, LC-MS and TLC-DPPH^•^ bioautography/MS analyses were performed to elucidate the structures of the degradation products.

To reduce the interruption of pH during the cellular tests, the acidic and alkaline hydrolysis samples were neutralized prior to the initial dilution of the sample. However, the LC profiles were altered by neutralization, which suggested that some of the by-products were produced by neutralization.

In pharmacokinetics and decomposition studies, hydrolysis of the ester linkages of the iridoid valepotriates readily produced baldrinals [33]. In the stress tests, baldrinal was detected as a degradation product and showed reduced antioxidant activities. The main reacting compounds might play critical roles as donating electron centres. A study by Marc et al. demonstrated that the oxirane nucleus was important for exerting anti-proliferative activities in neuroblastoma cells, and our work agrees with the literature [38–40].

**Fig 5:**
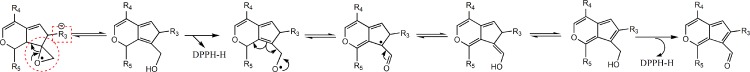
Proposed mechanism for the DPPH^•^ reaction.

Based on the forced degradation data, the donation of two hydroxyl groups from the oxirane nucleus was deduced as a possible antioxidant mechanism. The reactions shown in Fig 5 are example DPPH^•^ reactions. The oxirane ring could generate hydroxyl groups through the intermolecular transfer of an H^•^, which could interact with the oxidation reagent [41]. The acetoxy group from R_3_ (Fig 5) possesses a conjugated group in the ortho position, which might enter into resonance with the oxirane ring. The acetoxy group from R_5_ would therefore be delocalized outside of the oxirane ring, and this action might lower the chance of complex derivation. This effect could explain why AV showed stronger antioxidant activity than BAV in our study.

A second hydroxyl group is regenerated through the intramolecular transfer of a successive H^•^ and could once again interact with DPPH^•^. Aromatic rings with unsaturated chains are essential for both finger root and valerian bioactivities [33, 42]. The poor efficiency of the degradation products and the slow DPPH^•^ colour development reactions might be explained by the absence of electron-donating groups [43].

Our work also indicates that iridoid valepotriates can significantly scavenge free radicals *in vitro*, alleviate ROS levels and induce apoptosis in HepG2 cells. Another possible mechanism to explain the ROS resistance in cancer cells was elucidated by combining the results of our cancer cells experiments with information from the literature [44–47]. GABA is an important amino acid that mainly serves as an inhibitory neurotransmitter in the CNS [40]. It is generally accepted that valerian might reduce oxidant effects by modulating γ-amino butyric acid A receptor (GABA_A_R)-mediated signalling. Previous studies have shown that valepotriates bind to GABA_A_R at benzodiazepine (BzD) receptor site, thereby activating the receptor [45]. The overall increase in GABA_A_R activity was shown to inhibit the proliferation of HepG2 cells [46].

**Fig 6:**
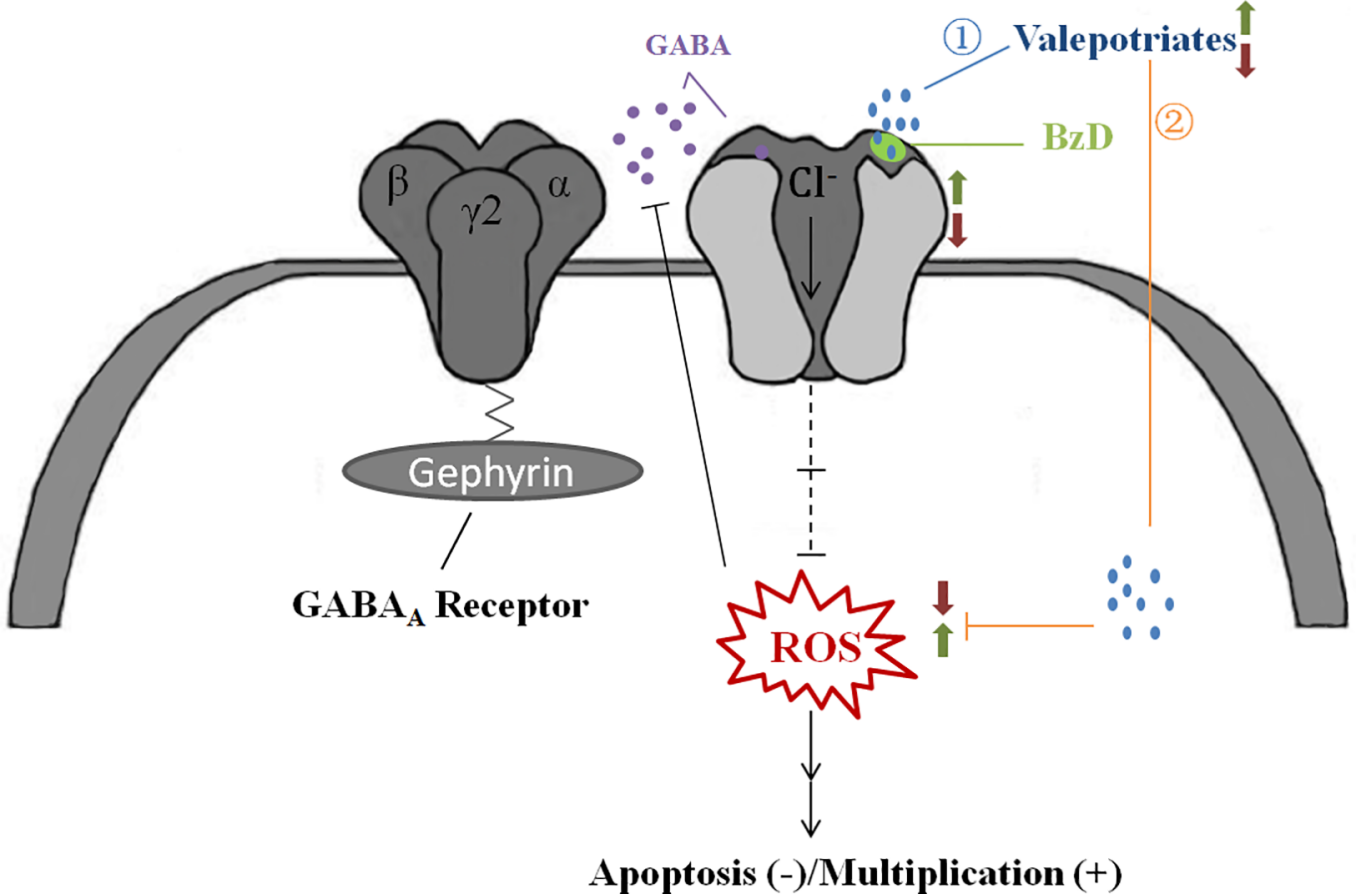
Proposed interactions between valepotriates and the GABAergic signalling pathway.

In neuropathic pain studies, excessive ROS levels might induce pain by reducing synaptic GABA release and thereby interfering with GABA-mediated inhibitory transmission [46]. In other words, increased ROS levels can decrease the synaptic release of GABA [47, 48]. HepG2 cells were studied in this work due to their sensitivity in the MTT test. The drugs used in all of the tests suppressed the ROS levels and inhibited proliferation. Thus, the results suggest that iridoid valepotriates might play a role in GABAergic signalling pathways (Fig 6) [49]. Herein, valerian is important in oxidant resistance and neuroprotective activities.

In summary, our work demonstrates that iridoid valepotriates exert effective antioxidant effects. However, additional studies are needed to further explore the interactions between iridoid valepotriates and the GABAergic signalling pathway. Moreover, epidemiological studies should be undertaken to discover whether iridoid valepotriates can be exploited as effective antioxidant and neuroprotective agents.

## Supporting information

S1 Fig HSBC spectrum. (A) AV HMBC spectra for groups 1 (H-1, C-22) and 2 (H7, C-16). (B) BAV HMBC spectra for groups 3 (H-1, C-22) and 4 (H7, C-16).
(TIF)

S2 Fig TOF/MS spectra for the [M+Na]^+^ ion and the plausible acevaltrate fragmentation pathway. (TIF)

S3 Fig **TLC-DPPH chromatography of (A) V, (B) AV and (C) BAV.** Lines 1–6 show the results obtained after 60°C for 0 h (control), 60°C for 1 h, 60°C for 2 h, 60°C for 4 h, 60°C for 6 h, and 25°C for 7 days, respectively.
(TIF)
