# Supplementary figures and images for: Studies of the structure-antioxidant activity relationships and antioxidant activity mechanism of iridoid valepotriates and their degradation products
Source: PLoS One. 2017 Dec 12;12(12):e0189198. doi: 10.1371/journal.pone.0189198 (PMC5726618; doi:10.1371/journal.pone.0189198)

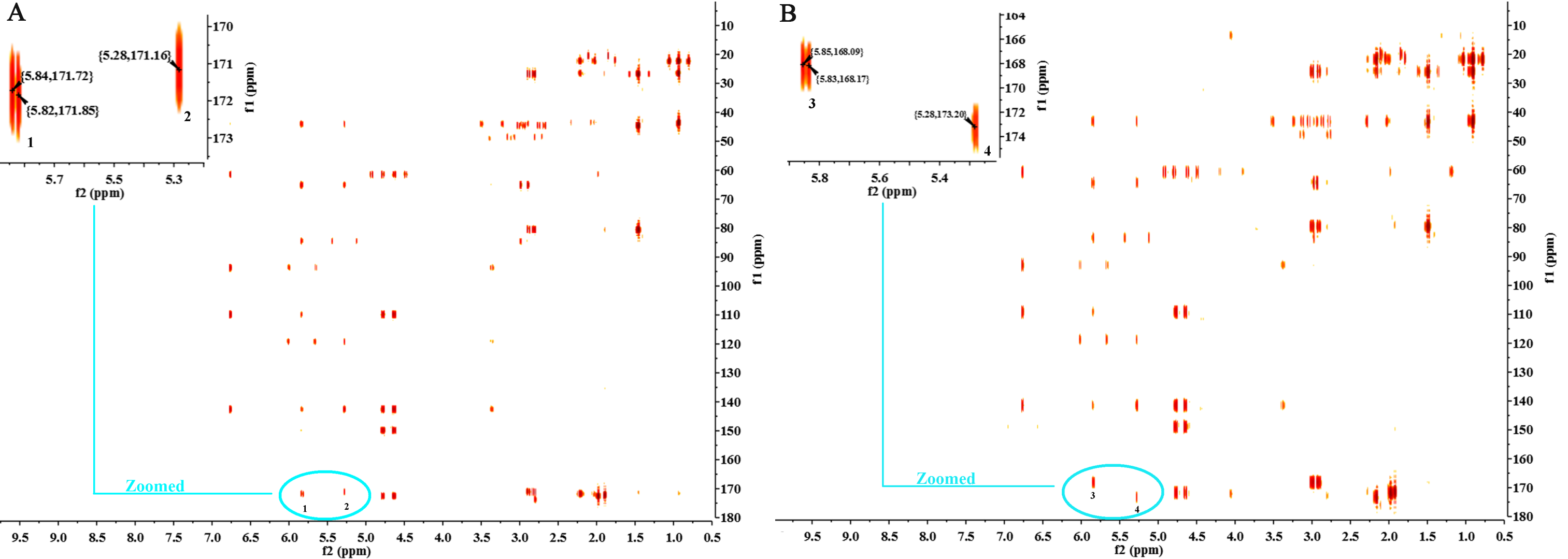

Supplement: S1 Fig — (A) AV HMBC spectra for groups 1 (H-1, C-22) and 2 (H7, C-16). (B) BAV HMBC spectra for groups 3 (H-1, C-22) and 4 (H7, C-16). (TIF) [file pone.0189198.s001.tif]

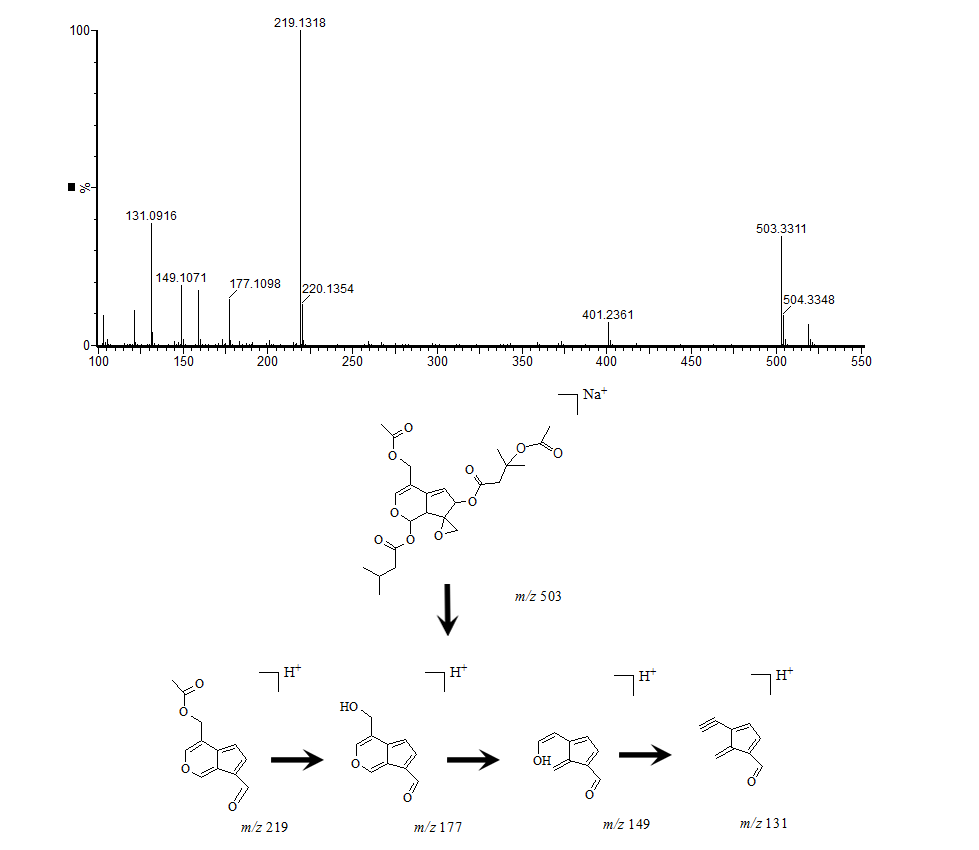

Supplement: S2 Fig — (TIF) [file pone.0189198.s002.tif]

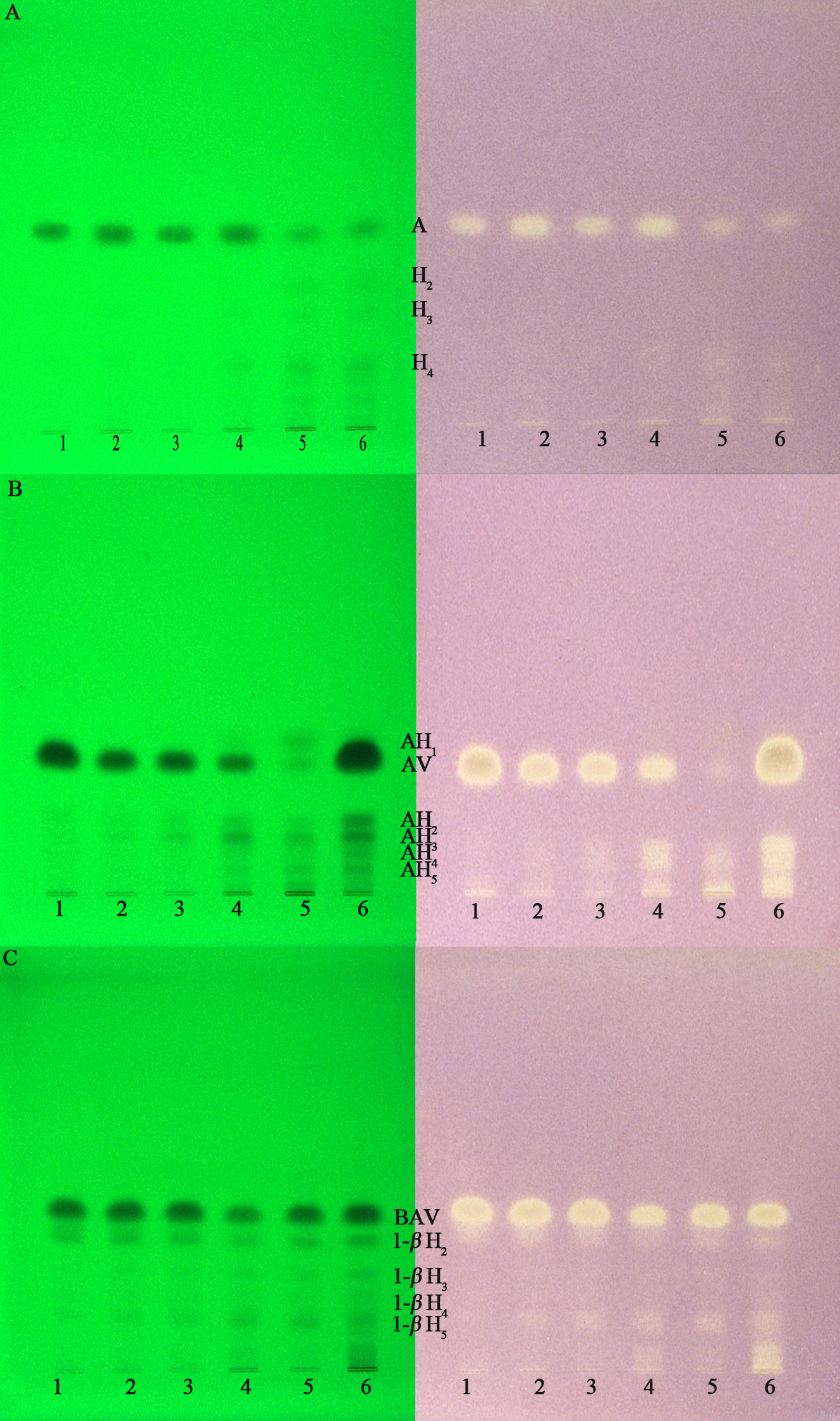

Supplement: S3 Fig — TLC-DPPH chromatography of (A) V, (B) AV and (C) BAV. Lines 1–6 show the results obtained after 60°C for 0 h (control), 60°C for 1 h, 60°C for 2 h, 60°C for 4 h, 60°C for 6 h, and 25°C for 7 days, respectively. (TIF) [file pone.0189198.s003.tif]
